# When does narcissism matter? Motivation and peer interaction as conditional pathways to social loafing in college physical education

**DOI:** 10.3389/fpsyg.2026.1790033

**Published:** 2026-04-20

**Authors:** Xiao-Lei Xi, Xin-Mao Lin, Mei-Yao Huang, Kuo-Tung Shih

**Affiliations:** 1 Luoyang Vocational College of Culture and Tourism, Luoyang, China; 2 Department of Physical Education, Huaiyin Normal University, Huai'an, Jiangsu, China; 3 Graduate Institute of Physical Education, National Taiwan Sport University, Taoyuan, Taiwan; 4 Indigenous Program of Sport Promotion, National Taiwan Sport University, Taoyuan, Taiwan

**Keywords:** motivation, narcissism, peer interaction, physical education, social loafing, sport psychology

## Abstract

**Objective:**

Drawing on social cognitive theory, this study examined the moderating role of narcissism in the relationships among motivation, peer interaction, and social loafing in college physical education settings.

**Methods:**

A total of 454 college students from Taiwan and China completed measures assessing motivation in physical education, peer interaction, narcissistic personality traits, perceived exertion during a team task, and cardiovascular fitness performance. Social loafing was operationalized using perceived exertion in conjunction with fitness performance. Moderated hierarchical regression analyses were conducted to examine the interaction effects, followed by simple slope analyses to probe significant interactions.

**Results:**

Bivariate analyses indicated that narcissism was positively associated with motivation, positive peer interaction, and perceived exertion during team tasks. Moderated regression analyses revealed that narcissism significantly moderated the relationships between motivation, positive peer interaction, and social loafing. Specifically, the strength of the associations between motivation, positive peer interaction, and perceived exertion differed across levels of narcissism. Negative peer interaction was associated with poorer cardiovascular fitness performance.

**Conclusion:**

These findings suggest that narcissism functions as a conditional factor shaping how motivational and social processes translate into individual effort in team-based physical education contexts. Rather than exerting a uniform effect, narcissism alters the strength of the associations among motivation, peer interaction, and social loafing. The results underscore the importance of considering personality traits when designing instructional strategies to reduce social loafing and promote active engagement in physical education.

## Introduction

Social loafing is a well-established concept in research on group performance and teamwork (Naeimi and Azimkhani, 2022; Woodman et al., 2011; Williams et al., 1981; Huang et al., 2019). It refers to the tendency for individuals to reduce their personal effort when working in a group compared with situations in which their individual contributions are directly identifiable (Latané et al., 1979; Woodman et al., 2011). This phenomenon has often been discussed in relation to the Ringelmann effect, which describes the decline in individual effort as group size increases during collective tasks (Ingham et al., 1974; Karau and Williams, 1993). Group-based physical activities provide a context in which social loafing may occur because task responsibilities are shared among team members (Karau and Williams, 1993; Heuzé and Brunel, 2003; Woodman et al., 2011; Czyż et al., 2016; Swain, 1996). In sport and physical education settings, this phenomenon has been frequently observed in cooperative or team-based exercise tasks where individual effort may be less visible (Huang et al., 2019; Woodman et al., 2011). As a result, social and motivational dynamics within group environments may influence how individuals regulate their effort during physical activity tasks. In school physical education (PE), many sports activities are organized as team-based tasks that require interaction among students. Peer interaction in such contexts may involve both positive experiences (e.g., encouragement and cooperation) and negative experiences (e.g., conflict or lack of support), which may differentially influence students’ motivation and effort during group activities (Chidambaram and Tung, 2005; Ward and Lee, 2005). Previous research has therefore suggested that interpersonal dynamics within groups may play an important role in shaping effort regulation and the emergence of social loafing in physical education contexts.

Building on the role of group dynamics in the emergence of social loafing, peer interaction represents an important social factor that may influence students’ motivation and effort regulation in physical education contexts. Peer interaction in physical education may involve both positive and negative experiences. Positive peer interaction, such as encouragement, cooperation, and mutual support, may promote engagement and sustained effort in team-based activities (Ryan, 2001; Wentzel, 1998). In contrast, negative peer interaction, including conflict, competition, or lack of cooperation, may undermine motivation and increase the likelihood of reduced effort or disengagement in group-based tasks (Juvonen and Wentzel, 1996; Ryan, 2001). Consistent with social cognitive perspectives, motivational and social cues within group environments may therefore influence individuals’ effort regulation during collective tasks in school physical education (Naeimi and Azimkhani, 2022; Huang et al., 2019; Czyż et al., 2016; Swain, 1996).

In addition to social influences, individual personality characteristics may also shape how students respond to group-based motivational environments. One such characteristic is narcissism. Narcissism is generally characterized by a strong desire for admiration, heightened self-confidence, and a tendency to pursue opportunities for self-enhancement (American Psychiatric Association, 2013; Tuckman and Sexton, 1992; Wallace and Baumeister, 2002). Previous studies have shown that individuals with higher levels of narcissism tend to view themselves as superior to others and are motivated to demonstrate their abilities and achievements (Campbell et al., 2004; Farwell and Wohlwend-Lloyd, 1998; Gabriel et al., 1994; John and Robins, 1994; Wallace and Baumeister, 2002). Because narcissistic individuals are particularly sensitive to opportunities for recognition and performance visibility, they may be especially responsive to motivational and social cues in group environments. Research in sport and performance contexts suggests that narcissistic individuals may increase their effort when group situations provide opportunities for self-enhancement or performance visibility (Woodman et al., 2011; Huang et al., 2019; Wallace and Baumeister, 2002). Taken together, these findings suggest that narcissism may shape how individuals respond to motivational and interpersonal dynamics within team-based physical activities. Specifically, narcissism may strengthen the influence of motivational and peer interaction factors on effort regulation, thereby affecting the likelihood of social loafing in group-based physical education tasks.

Numerous theoretical perspectives suggest that narcissistic characteristics may influence individuals’ effort regulation in group contexts. Because narcissistic individuals tend to pursue opportunities for self-enhancement and recognition, they may strive to outperform others during performance tasks. Consequently, narcissism has been proposed as a personality factor that could reduce social loafing when individuals seek to demonstrate their superiority in group-based physical activities. However, empirical findings regarding the relationship between narcissism, performance, and social loafing have been inconsistent. For example, Woodman et al. (2011) found that narcissists’ performance significantly improved when their contributions were identifiable within a group task, whereas non-narcissistic individuals showed no such differences. Similarly, Wallace and Baumeister (2002) reported that narcissists performed better when group tasks provided opportunities for self-enhancement. Nevertheless, other studies suggest that although narcissistic individuals often perceive themselves as superior performers, their actual performance may not differ significantly from that of others (Woodman et al., 2011; Huang et al., 2019). Taken together, these findings suggest that narcissistic individuals may be particularly motivated when group contexts provide opportunities for visibility and recognition. Despite growing interest in narcissism in performance contexts, relatively little research has examined how narcissism operates in physical education environments, particularly during group-based physical activities where peer interaction and motivation play important roles. School physical education provides a natural setting for examining how personality characteristics interact with social and motivational factors during collective physical tasks. Previous research has shown that peer interaction and motivational climates are associated with performance and engagement in physical education contexts (Huang et al., 2019). However, to the best of our knowledge, few studies have directly examined how narcissism may shape the relationships among peer interaction, motivation, and social loafing in school physical education settings. Therefore, the present study aims to investigate the role of narcissism in shaping effort regulation during team-based physical activities in school physical education. Specifically, this study examines whether narcissism moderates the relationships between peer interaction, motivation, and social loafing in group physical tasks.

Based on previous studies, it is apparent that motivation and peer interaction can influence narcissism to strive for challenging tasks and perform under pressure in the physical education context (Woodman et al., 2011; Huang et al., 2019). Furthermore, previous research supports the notion that narcissism can contribute to social loafing, although the moderating effect of narcissism has not been considered (Woodman et al., 2011; Huang et al., 2019). Additionally, the relationship between narcissism, motivation, peer interaction, and their influence on the RPE in group tasks remains unclear. Overall, studies examining these relationships have emphasized the need to identify whether narcissism has unique effects on social loafing within the field of physical education. By doing so, narcissism may influence how motivation and peer interaction relate to effort in group-based PE tasks involving cardiovascular fitness performance, leading individuals to invest greater effort in physical education activities. Therefore, grounded in social cognitive theory, the present study aimed to examine the moderating role of narcissism in the associations among motivation, peer interaction, and social loafing in college physical education (PE) contexts. Specifically, this study examined whether narcissistic personality traits moderate the relationships between students’ motivation in physical education, perceived peer interaction, and social loafing during team-based physical activities. We made the following hypothesis:

*H1*: Motivation in PE and positive peer interaction will be negatively associated with social loafing, whereas negative peer interaction will be positively associated with social loafing.

*H2*: Narcissism will moderate the relationship between motivation in PE and social loafing, such that the negative association between motivation and social loafing will be stronger among individuals with higher levels of narcissism.

*H3*: Narcissism will moderate the associations between positive peer interaction, negative peer interaction, and social loafing.

## Methods

### Participants

A total of 454 participants were recruited from physical education classes across several regions in East Asia, including Taipei City (*N* = 90, 19.80%), New Taipei City (*N* = 89, 19.40%), Henan Province (*N* = 92, 20.30%), Hebei Province (*N* = 91, 20.00%), and Beijing (*N* = 93, 20.50%). Participants were recruited during the data collection period, and all students who met the inclusion criteria and agreed to participate were included in the final sample. To be eligible to participate in the study, participants had to meet the following inclusion criteria: (a) be between 18 and 24 years of age, corresponding to the developmental stage of emerging adulthood typically represented in undergraduate student populations, and (b) report no medical or physical conditions that could interfere with participation in the physical activity assessments. Three exclusion criteria were applied in this study: (a) participants reporting psychological or neurological conditions (e.g., emotional disorders or attention deficit hyperactivity disorder), (b) participants reporting sensory impairments (e.g., visual or hearing problems), and (c) participants reporting physical conditions that might limit participation in the physical fitness assessment (e.g., cerebral palsy, stroke, arthritis, spinal cord injury, epilepsy, or muscular dystrophy). These conditions were assessed using a self-reported health questionnaire completed prior to participation. Additionally, participants had the right to withdraw from the study at any time without facing any consequences. The average age of the participants was 19.55 years (SD = ±1.35), consisting of 242 males (53.30%), and 212 females (46.70%). The participants had an average height of 168.46 cm (SD = 9.11) and an average weight of 62.25 kg (SD = 11.80). The study protocol was approved by the Institutional Review Board of the University of Taipei (IRB-2018-013). All participants provided written informed consent prior to participation.

### Measures

#### Socio-demographic characteristics

##### Motivation in PE

The Motivation in PE Scale was developed by Chou et al. (2015) and is based on the expectancy-value theory (Song and Keller, 2001). The scale aims to assess individuals’ motivation in the context of PE and comprises four perceived behavior and mental components: attention, relevance, confidence, and satisfaction. The MPES consists of 12 items, each rated on a 5-point scale ranging from 1 (Not true) to 5 (Very true). To provide examples of the scale items, attention is measured by statements such as “This PE lesson has things that stimulated my curiosity.” Relevance is measured by items such as “I believe that the information available/provided in this PE class is important to me.” Confidence is assessed through statements like “A good skill demonstration helped me feel confident that I would learn/acquire this skill.” Satisfaction is measured by items like “It felt good to complete this motor skill successfully.” In a previous study conducted by Chou et al. (2015), factor analysis confirmed the four-factor structure of the Chinese version of the MPES, consistent with the theoretical expectations. The model fit indices were as follows: χ2/df = 2.76, GFI = 0.91, AGFI = 0.90, RMSEA = 0.07, SRMR = 0.01, and CFI = 0.98. Additionally, the composite reliability of the latent variables ranged from 0.88 to 0.91, indicating good internal consistency.

##### Peer interaction in PE scale

The Peer Interaction in PE Scale was developed by Chou et al. (2015) and assesses students’ perceptions of positive and negative interactive behaviors among peers in a physical education class (Coie et al., 1982). The scale consists of 24 items that capture various aspects of peer interaction. The positive peer interaction component comprises three sub-dimensions: cooperative behavior, game participation behavior, and respectful behavior. These dimensions reflect an individual’s satisfaction with how they interact with others. The negative peer interaction component also consists of three sub-dimensions: dominance, conflict, and jealousy. These dimensions capture inappropriate ways of communicating with others. Participants rate their perceptions of peer interaction in PE on a 5-point scale, ranging from 1 (Never) to 5 (Always). For instance, cooperative behavior is measured with items like “I feel good cooperating with other classmates while participating in game [*sic*] activity.” Game participation behavior is assessed with items such as “I am willing to participate in a game with others.” Respectful behavior is measured through statements like “I believe that my respectful behaviors positively influence others’ efforts to gain better performance.” On the other hand, dominance is measured with items like “Sometimes it is necessary to step on others in the class to get ahead of them in motor skill performance.” Conflict is captured through statements such as “It is sometimes necessary to use fighting against others to get what I want in PE class.” Jealousy is assessed with items like “No matter how hard I try, my teacher seems to be more interested in others’ performance than mine.” Moreover, the conformity factor analysis results of the Peer Interaction in PE Scale has six factors, as theoretically expected, and the model fit indices were as follows: χ^2^/df = 1.43, GFI = 0.95, AGFI = 0.92, RMSEA = 0.04, SRMR = 0.01, and CFI = 0.99, while the composite reliability of latent variables ranged from 0.91 to 0.93 (Chou et al., 2015).

#### Narcissistic personality inventory

Narcissism was measured using the Narcissistic Personality Inventory (NPI), developed by Raskin and Terry (1988), which is widely used to assess grandiose narcissism in non-clinical populations. The NPI is a self-report instrument consisting of 40 forced-choice items designed to assess narcissistic traits among college students. It captures seven components of narcissism: authority, exhibitionism, superiority, vanity, exploitativeness, entitlement, and self-sufficiency. For each item, participants choose between two statements, one reflecting a narcissistic trait and the other reflecting a non-narcissistic attitude. Narcissistic responses are scored as 1 and non-narcissistic responses as 0, and the total narcissism score is obtained by summing the endorsed narcissistic responses. Examples of NPI items include the following:

1 A: “I am not sure if I would make a good leader.”

B: “I see myself as a good leader.”

2 A: “If I feel competent, I am willing to take responsibility for making decisions.”

B: “I like to take responsibility for making decisions.”

The NPI has demonstrated satisfactory internal consistency for its component scales, with Guttman lambda 3 and alpha coefficients ranging from 0.50 to 0.83. It also exhibits factorial validity, as indicated by correlations (ranging from 0.24 to 0.44) among the component scales. Previous validation studies of the 40-item NPI have reported satisfactory reliability, with Cronbach’s *α* typically ranging from approximately 0.80 to 0.90 in non-clinical samples (Raskin and Terry, 1988). Moreover, the NPI has shown construct validity, with 52% of the variance accounted for by narcissism. In the present sample, the Narcissistic Personality Inventory demonstrated acceptable internal consistency (Cronbach’s *α* = 0.82).

#### Social loafing

Following the approach suggested by Woodman et al. (2011), social loafing in the present study was operationalized using indicators reflecting individual effort during team-based physical tasks. In physical performance contexts, reductions in individual effort within group tasks can be inferred from the relationship between physiological performance and perceived exertion. Therefore, social loafing was indexed by combining cardiovascular fitness performance and the rating of perceived exertion (RPE) during the team task. The rating of perceived exertion was measured using the Borg RPE scale (Borg, 1998), which ranges from 6 (“very, very light exertion”) to 20 (“maximal exertion”). In the present study, RPE represented participants’ perceived level of effort while performing the team-based physical activity in physical education. Higher RPE scores indicate greater perceived effort and therefore lower levels of social loafing, whereas lower RPE scores indicate reduced effort and greater social loafing. Cardiovascular fitness performance was assessed using an adapted protocol from the American College of Sports Medicine guidelines (American College of Sports Medicine, 2014). Participants completed a 1,600-m run for men and an 800-m run for women as fast as possible. Running time was recorded in seconds, with shorter completion times indicating better performance. The raw scores were subsequently converted into sex-specific standardized T-scores to allow comparison across participants. Accordingly, the composite index reflects individual effort, such that higher scores indicate lower levels of social loafing.

### Procedure

Before conducting data collection, the researchers took four steps to ensure proper communication and informed consent from the participants. Initially, the directors of the Departments of Student Affairs were contacted through email and phone calls to explain the purpose of the research, as well as to provide a brief overview of the confidentiality and anonymity measures in place. Once an agreement was reached, appointments were scheduled for data collection. Therefore, the study procedure consisted of four stages: (a) confirmation, (b) questionnaire, (c) assessment of cardiovascular fitness performance, and (d) assessment of perceived exertion for efforts on a team task in PE. During the confirmation stage, which took place during the spring and fall semester of 2022, participants completed a consent form and a self-reported health questionnaire that included questions about medical conditions, physical limitations, or disabilities that might affect their participation in the physical fitness assessment. During this process, participants were fully informed about the study and its objectives. Each participant then entered the questionnaire stage, during which the survey package was administered. The survey package provided to the participants included a demographic questionnaire, along with the Motivation in PE Scale, Peer Interaction in PE Scale, and Narcissistic Personality Inventory. To minimize the potential influence of social desirability bias, the researchers made sure to inform the participants about the nature of the study. Specifically, they emphasized that the research aimed to explore college students’ experiences in PE, and that there were no right or wrong answers. Additionally, participants were assured that all responses would be treated as confidential. On the same day, we collected anthropometric data (i.e., age, height, weight, PA days per week, and PA times per day) from each participant. On the following day, during the cardiovascular fitness assessment stage, participants were randomly assigned to teams of five members. They were instructed to perform the running task as part of a team-based activity, in which their effort contributed to the overall team performance. Each participant was asked to run as fast as possible while completing the assigned distance. Although individual running times were recorded in seconds, participants were informed that their performances would contribute to the evaluation of the team task. Immediately after completing the running task, participants reported their rating of perceived exertion (RPE) regarding their effort during the team activity in physical education.

### Data analysis

Prior to conducting the statistical analyses, composite scores for the Motivation in PE Scale and the Peer Interaction in PE Scale were computed by averaging the corresponding subscale scores, with higher values indicating greater levels of the respective constructs. A three-step hierarchical moderated regression analysis was conducted to examine the moderating effect of narcissism on the relationships among motivation in physical education (PE), peer interaction in PE, and social loafing among college students. Similar analytical approaches have been used in previous studies examining moderating effects (Ahmetoglu et al., 2015; Balkis and Duru, 2017). Prior to the analyses, social loafing was operationalized as a composite index combining standardized cardiovascular fitness performance (T-scores) and ratings of perceived exertion during the team task. In Step 1, socio-demographic characteristics (gender, age, height, weight, body mass index, PA days per week, and PA times per day) were entered as control variables. In Step 2, the main effects of motivation in PE, positive peer interaction, negative peer interaction, and narcissism were entered into the model. In Step 3, three two-way interaction terms were added to test the moderating role of narcissism: narcissism × motivation, narcissism × positive peer interaction, and narcissism × negative peer interaction. The change in *R*^2^ at Step 3 reflects the combined contribution of these interaction terms to the model. Moderation analyses were conducted using PROCESS v3.4 for SPSS (Hayes, 2017). For significant interaction effects, simple slope analyses were conducted at low (−1 SD) and high (+1 SD) levels of narcissism to facilitate interpretation of the moderating effects.

## Results

### Comparisons of study variables by different levels of narcissism

To facilitate descriptive interpretation of the sample characteristics, participants were grouped into relatively higher and lower levels of narcissism using a median split. This grouping was used only for descriptive comparisons in Table 1 and was not used in the moderation analyses, which were conducted using continuous variables. As shown in Table 1, the two groups did not significantly differ in age, height, weight, BMI, physical activity days per week, or previous semester PE scores [*t*_(452)_ = −1.41, −1.47, −0.62, 0.21, −1.37, and –1.64, *ps* >0.05]. However, the groups differed significantly in physical activity frequency and duration [*t*_(452)_ = −4.58, *p* < 0.05]. However, there was no significant difference between gender and different levels of narcissism (*χ*^2^ = −0.43, *p* > 0.05).

**Table 1:** Comparison of socio-demographic characteristics between high and low narcissism groups (median split).

Variable	Narcissism				*t*	*χ* ^2^	*p*
	High	Low					
	Mean/n	SD/%	Mean/n	SD/%			
Gender							
Men	107	52.20	135	54.20		−0.43	0.67
Women	98	47.80	114	45.80			
Age	19.65	1.44	19.47	1.27	−1.41		0.16
Height	169.15	9.58	167.89	8.70	−1.47		0.14
Weight	64.63	12.64	63.94	11.09	−0.62		0.54
BMI	22.59	4.31	22.66	3.54	0.21		0.83
PA days per week	3.35	2.77	2.91	3.93	−1.37		0.17
PA times per day	50.37	31.93	37.62	27.35	−4.58*		0.01

*p < 0.05.

### Bivariate correlation analyses

The Pearson correlation analysis revealed that narcissism was significantly positively associated with motivation in physical education, positive peer interaction, and perceived exertion during the team task (*r* = 0.49, 0.62, and 0.42, respectively, *p*s < 0.01). In contrast, narcissism was negatively associated with negative peer interaction and cardiovascular fitness performance (*r* = −0.39 and –0.11, respectively, *ps* < 0.01). These findings suggest that students with higher levels of narcissism tended to report greater motivation, more positive peer interactions, and higher perceived effort during team tasks in physical education, while showing lower cardiovascular fitness performance and lower levels of negative peer interaction. In addition, the correlations between each subscale of negative peer interaction (dominance, conflict, and jealousy) and social loafing showed patterns consistent with the composite measure. Specifically, dominance (*r* = −0.16), conflict (*r* = −0.15), and jealousy (*r* = −0.20) were all negatively associated with social loafing, similar to the composite variable (*r* = −0.18). Because higher scores on the social loafing index reflect greater individual effort (i.e., lower levels of actual social loafing behavior), positive correlations with this variable indicate reduced social loafing, whereas negative correlations indicate increased social loafing. Thus, the direction of the observed correlations should be interpreted relative to the construct of social loafing rather than the raw coding of the composite index. Accordingly, the positive correlations of motivation and positive peer interaction with the social loafing index, and the negative correlation of negative peer interaction, are consistent with the hypothesized directions in H1. Table 2 presents the bivariate correlations among study variables.

**Table 2:** Correlation analysis of students’ narcissism, motivation in physical education (PE), peer interaction, cardiovascular fitness, perceived exertion on a team task, and social loafing.

**Variables**	**1**	**2**	**3**	**4**	**5**	**6**	**7**	**8**	**9**	**10**	**11**	**12**	**13**	**14**	**15**	**16**	**17**
1. Narcissism	~																
2. Motivation in PE	0.49**	~															
3. Attention	0.43**	0.89**	~														
4. Relevance	0.41**	0.91**	0.76**	~													
5. Confidence	0.50**	0.93**	0.74**	0.81**	~												
6. Satisfaction	0.43**	0.88**	0.69**	0.69**	0.77**	~											
7. Positive peer interaction	0.62**	0.45**	0.40**	0.37**	0.43**	0.42**	~										
8. Cooperative behavior	0.63**	0.44**	0.39**	0.37**	0.43**	0.41**	0.98**	~									
9. Game participation behavior	0.60**	0.44**	0.39**	0.36**	0.43**	0.42**	0.98**	0.93**	~								
10. Respectful behavior	0.59**	0.43**	0.39**	0.36**	0.41**	0.39**	0.97**	0.92**	0.92**	~							
11. Negative peer interaction	−0.38**	−0.25**	−0.20**	−0.26**	−0.24**	−0.19**	−0.45**	−0.44**	−0.44**	−0.43**	~						
12. Dominance	−0.39**	−0.22**	−0.17**	−0.24**	−0.20**	−0.16**	−0.36**	−0.36**	−0.35**	−0.34**	0.88**	~					
13. Conflict	−0.31**	−0.23**	−0.17**	−0.24**	−0.23**	−0.17**	−0.45**	−0.43**	−0.45**	−0.44**	0.94**	0.72**	~				
14. Jealousy	−0.35**	−0.25**	−0.22**	−0.26**	−0.23**	−0.19**	−0.43**	−0.42**	−0.42**	−0.41**	0.94**	0.73**	0.87**	~			
15. Cardiovascular fitness	−0.11*	−0.13**	−0.13**	−0.13**	−0.11*	−0.12*	−0.14**	−0.13**	−0.13**	−0.14**	0.14**	0.12**	0.14**	0.11*	~		
16. Perceived exertion on a team task	0.42**	0.32**	0.30**	0.28**	0.31**	0.26**	0.34**	0.37**	0.32**	0.31**	−0.31**	−0.28**	−0.27**	−0.30**	−0.27**	~	
17. Social loafing	0.30**	0.18**	0.17**	0.15**	0.19**	0.14**	0.21**	0.24**	0.20**	0.18**	−0.18**	−0.16**	−0.15**	−0.20**	0.41**	0.76**	~
Mean	13.51	3.10	3.08	3.15	3.03	3.13	3.61	3.61	3.57	3.66	2.38	2.64	2.10	2.38	240.89	14.72	724.95
Stand deviation	6.89	0.75	0.82	0.82	0.87	0.84	1.23	1.24	1.29	1.27	1.02	1.04	1.23	1.06	37.25	4.14	222.11

*p < 0.05; **p < 0.01; The social loafing variable is coded such that higher scores reflect greater individual effort (i.e., lower levels of actual social loafing behavior). Therefore, positive correlations indicate reduced social loafing.

### Test of the moderation model of narcissists

A three-step analysis of moderated hierarchical regression was used to examine the moderating effect of narcissism on relationship motivation and peer interaction on social loafing. In the first step, the control variables were analyzed on the socio-demographic characteristics (e.g., gender, age, height, weight, BMI, PA days per week, and PA times per day). The moderated hierarchical regression analysis indicated a significant model [*F*_(7, 446)_ = 2.12, *p* < 0.05, *R^2^* = 0.03, *∆R*^2^ = 0.02]. Moreover, social loafing was significantly associated with PA times per day in collegiate students (*ß* = −0.17, *t* = 3.42, *p* < 0.05). Gender did not significantly predict social loafing in the regression models (*ß* = –0.05, *t* = 0.80, *p* > 0.05), suggesting that the observed relationships were not driven by gender differences in the running task. In the second step, motivation in PE, positive peer interaction, negative peer interaction, and narcissism were entered into the model. The addition of these predictors significantly improved the fit of the model [*F*_(4, 442)_ = 9.72, *p* = 0.0001, *R*^2^ = 0.11, *∆R*^2^ = 0.09]. In this model, social loafing was significantly associated with PA times per day and narcissism among collegiate students (*ß* = 0.11 and 0.23, *t* = 2.23, and 3.70, *p* < 0.05). In the third step, social loafing was predicted by adding interactions of narcissism with, motivation in PE, positive peer interaction, and negative peer interaction among collegiate students. The inclusion of the interaction terms significantly improved the model fit [*F*_(4, 437)_ = 3.81, *p* = 0.02, *R^2^* = 0.13, Δ*R^2^* = 0.02]. The results also showed that PA times per day, motivation in PE, positive peer interaction, and negative peer interaction significantly contributed to social loafing (*ß* = 0.12, 0.29, −0.29, and –0.22, *t* = 2.30, 2.50, −2.33, and –1.97, *p* < 0.05). Of particular interest, the interaction between narcissism and motivation was statistically significant (*β* = −0.75, *t* = −2.41, *p* < 0.02), as was the interaction between narcissism and positive peer interaction (*β* = 0.81, *t* = 2.61, *p* < 0.01), indicating that narcissism moderated the associations between these variables and social loafing. In contrast, the interaction between narcissism and negative peer interaction was not statistically significant (*β* = 0.19, *t* = 1.36, *p* < 0.17).

**Figure 1:**
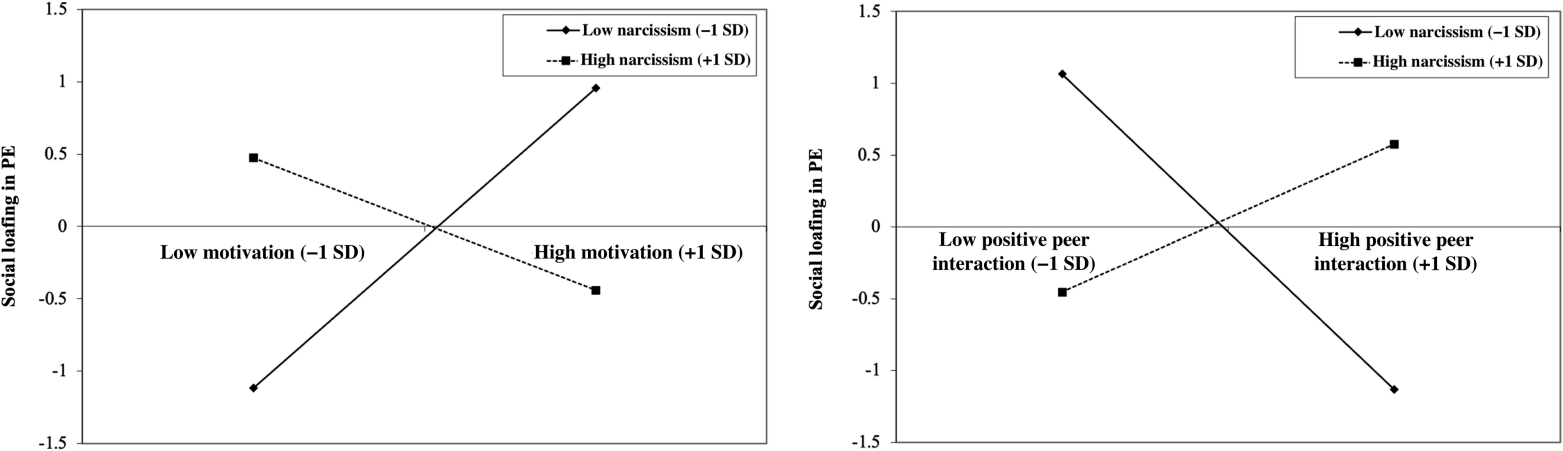
The effect of narcissism on the interaction between motivation, peer interaction, and social loafing.

Simple slope analyses were conducted to probe the significant interaction effects by estimating the regression slopes at low (−1 SD) and high (+1 SD) levels of narcissism. Because higher scores on the social loafing index reflect greater individual effort (i.e., lower levels of actual social loafing), the direction of the regression coefficients should be interpreted accordingly. Simple slope analyses indicated that when narcissism was high (+1 SD), motivation was negatively associated with the social loafing index (*B* = −0.46, SE = 0.21, *t* = −2.15, *p* < 0.05), indicating that higher motivation was associated with lower effort (i.e., greater social loafing). In contrast, when narcissism was low (−1 SD), the association between motivation and the social loafing index was positive and statistically significant (*B* = 1.035, SE = 0.416, *t* = 2.49, *p* < 0.01), indicating that higher motivation was associated with greater effort (i.e., lower social loafing). This pattern suggests that the direction of the relationship between motivation and social loafing reverses depending on the level of narcissism. As shown in Figure 1, the interaction between motivation and narcissism revealed a cross-over pattern. When motivation was low, higher (vs. lower) levels of narcissism were associated with greater social loafing, whereas when motivation was high, higher (vs. lower) levels of narcissism were associated with lower social loafing. Similarly, for positive peer interaction, simple slope analyses showed that when narcissism was high (+1 SD), positive peer interaction was positively associated with the social loafing index (*B* = 0.52, SE = 0.21, *t* = 2.46, *p* < 0.01), indicating that higher levels of positive peer interaction were associated with greater effort (i.e., lower social loafing). In contrast, when narcissism was low (−1 SD), the association between positive peer interaction and the social loafing index was negative (*B* = −1.10, SE = 0.42, *t* = −2.59, *p* < 0.01), indicating that higher levels of positive peer interaction were associated with lower effort (i.e., greater social loafing). This pattern also suggests that the direction of the relationship reverses depending on the level of narcissism. When interpreted at the construct level of social loafing, these findings indicate that the association between motivation and social loafing differs across levels of narcissism. Thus, although the moderating effect of narcissism is statistically significant, the observed cross-over pattern does not fully reflect the simple strengthening of a uniformly negative association as originally hypothesized in H2. Instead, the results suggest a more complex pattern in which the direction of the relationship varies across levels of narcissism. Taken together, these findings support the moderating role of narcissism in the associations between motivation, positive peer interaction, and social loafing, consistent with H3. However, the form of moderation differs from the original expectation in H2 and instead reflects a cross-over pattern.

## Discussion

The present study examined the moderating role of narcissism in the relationships among motivation, peer interaction, and social loafing in college physical education. Because the social loafing index was derived from effort-based indicators, higher scores represent greater individual effort (i.e., lower levels of social loafing), which should be considered when interpreting the direction of the associations. The present study examined how narcissism moderates the associations between motivation, peer interaction, and social loafing in physical education contexts. Consistent with social cognitive theory, the findings demonstrate that individual effort during team-based physical activities is shaped by the dynamic interplay between personal factors (narcissism), environmental factors (peer interaction), and behavioral indicators (perceived exertion). The results revealed that narcissism was positively associated with motivation, positive peer interaction, and perceived exertion during team tasks. However, narcissism did not exert a uniform direct effect on social loafing. Instead, moderated regression analyses indicated that the associations between motivation, positive peer interaction, and social loafing varied across levels of narcissism. Specifically, the association between motivation and social loafing differed across levels of narcissism, reflecting a cross-over pattern rather than a simple strengthening of a negative association. Notably, although the moderating effect of narcissism was statistically significant, the form of the interaction did not reflect a simple strengthening of the hypothesized negative association. Instead, the results revealed a cross-over pattern, indicating that the direction of the relationship between motivation and social loafing differs depending on the level of narcissism.

To further clarify this moderating effect, the results indicate that the relationship between motivation and social loafing varies depending on the level of narcissism. When narcissism is high, higher motivation was associated with lower effort (i.e., greater social loafing), indicating that motivation does not consistently translate into reduced social loafing under high narcissism. In contrast, when narcissism is low, motivation alone may be insufficient to sustain effort in group contexts. One possible explanation is that narcissistic individuals are particularly sensitive to opportunities for self-enhancement and recognition. When motivational and social cues signal that performance may be visible or valued, narcissistic individuals may increase their effort to maintain a favorable self-image and gain social recognition. These findings extend previous research by Woodman et al. (2011) by demonstrating that narcissism operates as a contextual amplifier within physical education settings. A similar moderating pattern was observed for peer interaction. However, contrary to expectations, at higher levels of positive peer interaction, higher narcissism was associated with greater social loafing. One possible explanation is that highly narcissistic individuals may strategically regulate their effort depending on opportunities for recognition and visibility. In supportive social environments, group members may experience reduced individual accountability, which may allow narcissistic individuals to conserve effort while still benefiting from positive group dynamics. Another possible explanation is that supportive social environments may reduce individual accountability in group tasks, allowing narcissistic individuals to conserve effort while still benefiting from positive group dynamics. Conversely, under less supportive motivational or social conditions, narcissism does not appear to provide the same advantage in reducing social loafing. Importantly, the present findings help clarify mixed results in the literature regarding narcissism and group performance. Rather than characterizing narcissism as inherently beneficial or detrimental, the current study suggests that its effects on social loafing are conditional on the motivational and social context of the task.

The present findings contribute to the ongoing debate regarding the role of narcissism in group-based performance contexts. These findings do not suggest that narcissism independently reduces social loafing. Rather, narcissism appears to influence how motivational and peer interaction factors relate to individual effort in group-based tasks. Although men and women completed different running distances (1,600 m vs. 800 m), gender was included as a control variable in the regression analyses and was not significantly associated with social loafing. This suggests that the observed relationships were unlikely to be driven by sex-specific differences in the running task. From a social cognitive theory perspective, narcissism functions as a personal factor that conditions how environmental and motivational cues are translated into behavioral effort during team-based physical activities. The results suggest that the effects of motivation and positive peer interaction are differentially regulated by individuals with elevated levels of narcissism, rather than uniformly reducing social loafing. Similar findings have been reported in previous studies exploring the relationship between narcissism, exercise motivation (Zeigler-Hill et al., 2021), peer interaction (Huang et al., 2019), and social loafing (Huang et al., 2019; Chou et al., 2015). Previous studies outlined a possible psychological mechanism to explain the performance of narcissists, emphasizing their enjoyment of self-enhancement opportunities and their ability to excel in challenging tasks within public settings (Woodman et al., 2011; Wallace and Baumeister, 2002). Furthermore, narcissists tend to exhibit perceived superiority and attract admiration in such situations where identifiability within a group task occurs, and they might increase on-task effort among narcissists in such a PE environment.

Notably, the findings also indicated that when narcissism was low, motivation was associated with greater social loafing. One possible explanation is that individuals with lower levels of narcissism may be less driven by self-enhancement and social recognition motives, which are important for translating motivation into observable effort in group contexts. In the absence of these self-presentational motives, motivation alone may not be sufficient to sustain individual accountability, particularly in team settings where responsibility is shared. Consequently, even when individuals report being motivated, this motivation may not consistently manifest as increased effort, resulting in higher levels of social loafing. This interpretation is consistent with a social-cognitive perspective, suggesting that personal traits condition how motivational cues are enacted behaviorally in group tasks. Overall, these findings suggest that narcissism shapes how motivational and social cues are translated into effort during group activities, rather than exerting a direct effect on social loafing. In terms of peer interaction, the interaction between narcissism and negative peer interaction was not statistically significant, suggesting that this aspect of peer dynamics may not be contingent on narcissistic tendencies in the present context. One possible explanation is that negative peer dynamics may operate more directly and be less dependent on individual personality traits such as narcissism.

According to Table 3, the present study found that individuals with higher levels of narcissism exhibited differential responses to motivational and social cues during group tasks in physical education. Specifically, while higher levels of narcissism strengthened the association between motivation and reduced social loafing, higher levels of positive peer interaction were associated with greater social loafing under conditions of high narcissism. This study contributes valuable information and insights into the moderating effect of narcissism on the relationship between motivation, peer interaction, and social loafing. Several possible mechanisms can explain how narcissism enhances the influence of motivation and peer interaction on social loafing. Firstly, based on theoretical grounds and narcissists’ self-appraisals, it is suggested that narcissists perceive their performance more favorably than others in group interaction tasks. Additionally, individuals with elevated levels of narcissism could make mediocre performers believe they have higher expectations of success, leading to actual performance improvement (Wallace and Baumeister, 2002). Secondly, the present investigation is guided by the theory on the effects of narcissism on performance. It is hypothesized that individuals with high levels of narcissism may lead to fluctuations in performance depending on situational opportunities and the rating of perceived exertion in PE. Furthermore, self-enhancement opportunities indicate the extent to which narcissists strive for excellent performance in team tasks to attain recognition and glory through perceived exertion in PE (Huang et al., 2019; Chou et al., 2015).

**Table 3:** Hierarchical multiple regression analysis to predict social loafing.

Variable	Step 1: direct effect			Step 2: direct effect			Step 3: interaction effects		
	*ß*	*t*	*p*	*ß*	*t*	*p*	*ß*	*t*	*p*
Gender	−0.05	−0.80	0.43	−0.01	−0.15	0.88	−0.01	−0.19	0.85
Age	0.02	0.52	0.61	0.01	0.10	0.92	0.00	0.02	0.98
High	0.42	1.46	0.15	0.25	0.88	0.38	0.31	1.13	0.26
Weight	−0.70	−1.44	0.15	−0.46	−0.96	0.34	−0.57	−1.21	0.23
BMI	0.62	1.39	0.17	0.40	0.92	0.36	0.48	1.11	0.27
PA days per week	−0.08	−1.54	0.12	−0.08	−1.60	0.11	−0.08	−1.60	0.11
PA times per day	0.17	3.42*	0.00	0.11	2.23*	0.03	0.12	2.30*	0.02
Motivation in PE				0.04	0.72	0.48	0.29	2.50*	0.01
Positive peer interaction				0.00	0.01	0.99	−0.29	−2.33*	0.02
Negative peer interaction				−0.08	−1.54	0.12	−0.22	−1.97*	0.05
Narcissism				0.23	3.70*	0.00	0.05	0.14	0.89
Narcissism × motivation in PE							−0.75	−2.41*	0.02
Narcissism × positive peer interaction							0.81	2.61*	0.01
Narcissism × negative peer interaction							0.19	1.36	0.17

*p < 0.05.

The findings of the present study diverged from previous research (Rose, 2007; Vazire and Funder, 2006). They reported that narcissists tend to excessively self-enhance and prioritize self-promotion over team performance in group tasks. However, Woodman et al. (2011) emphasized the significant role of narcissism in predicting team performance, suggesting that certain types of narcissism may be motivated to take on leadership roles in team tasks, particularly in the context of PE (Woodman et al., 2011). Previous studies have primarily focused on narcissists’ rating of perceived exertion during physical exercise, highlighting their tendencies to exhibit charismatic and leader-like attributes, such as an inflated sense of self and attention-seeking behavior (Nevicka et al., 2011). Taken together, these findings suggest that narcissism does not independently reduce social loafing. Instead, narcissism appears to shape how motivational and peer interaction factors are associated with individual effort in group-based contexts. In environments where motivation and positive peer interaction are present, narcissistic individuals may channel these cues into greater effort to maintain visibility and favorable self-evaluations. However, when such cues are weak, narcissism alone may be insufficient to prevent reductions in effort. It is also possible that broader cultural norms emphasizing group harmony and performance evaluation in educational settings influence how narcissistic traits are expressed during team-based physical activities. For example, in educational contexts where team performance contributes to course evaluation, individuals with higher levels of narcissism may be particularly motivated to demonstrate competence and achieve personal recognition during group tasks. Previous studies have similarly shown that narcissistic individuals often strive to outperform others when tasks provide opportunities for visibility and personal distinction (Wallace and Baumeister, 2002; Roberts et al., 2010).

This study has several limitations that should be considered when interpreting the results and planning future research. Firstly, future studies could explore the relationship between personal glory and social loafing by designing different intensities of cardiovascular exercise and varying the sizes of team members. This would allow for a more comprehensive understanding of how personal glory influences social loafing. Secondly, the cross-sectional nature of this study limits the ability to establish causality. It is unclear whether self-appraisals and personal glory are the primary causes of increased RPE, as the results may only indicate that narcissists’ motivation and peer interaction have an effect on social loafing. Additionally, while narcissists may exhibit self-enhancement tendencies in a PE context, previous research on actual performance quality has not consistently found benefits of narcissism (Wallace and Baumeister, 2002; Chou et al., 2015). Thirdly, this study only examined narcissism as a potential moderator, and other mediators such as self-esteem, leadership, group dynamics, or social support should be identified and tested in relation to the relationships between motivation, peer interaction, and social loafing in PE. Fourth, a potential limitation concerns the use of self-report measures for several variables, including motivation, peer interaction, narcissism, and perceived exertion. As these measures were collected within a similar time frame, common method bias cannot be entirely ruled out. Although procedural remedies were implemented (e.g., anonymity assurance and separation of questionnaire and performance assessments), future research could incorporate multiple data sources or longitudinal designs to further reduce potential method bias. Fifth, narcissism in the present study was assessed using the Narcissistic Personality Inventory. Because the NPI primarily measures grandiose narcissism, the present findings may not necessarily generalize to other forms of narcissism, such as vulnerable narcissism or other multidimensional conceptualizations of the construct. Future studies may benefit from examining whether different dimensions of narcissism show similar or distinct effects on social loafing in physical education contexts. Finally, although the sample size was relatively large, the participants consisted primarily of college students from a limited number of regions. Future studies including broader age groups and more diverse educational and cultural contexts may help improve the generalizability of the findings.

## Conclusion

This study contributes to a better understanding of the moderating role of narcissism in the relationships between motivation, peer interaction, and effort regulation during team-based physical activities in college physical education. The findings suggest that individuals with higher levels of narcissism tend to report higher motivation, greater positive peer interaction, and higher perceived exertion during team tasks. However, narcissism does not necessarily reduce social loafing; rather, it influences how motivational and interpersonal factors are associated with effort regulation in group contexts. In particular, narcissistic individuals may respond more strongly to social and motivational cues when group tasks provide opportunities for self-enhancement and performance visibility. These findings highlight the importance of considering personality characteristics, such as narcissism, when examining motivation, peer interaction, and effort regulation in team-based physical education activities.

## Data Availability

The original contributions presented in the study are included in the article/supplementary material, further inquiries can be directed to the corresponding author/s.
